# Partners coordinate territorial defense against simulated intruders in a duetting ovenbird

**DOI:** 10.1002/ece3.5821

**Published:** 2019-11-12

**Authors:** Pedro Diniz, Gianlucca S. Rech, Pedro H. L. Ribeiro, Michael S. Webster, Regina H. Macedo

**Affiliations:** ^1^ Programa de Pós‐Graduação em Ecologia Universidade de Brasília Brasília Brazil; ^2^ Departamento de Zoologia Laboratório de Comportamento Animal Universidade de Brasília Brasília Brazil; ^3^ Department of Neurobiology and Behavior Cornell Lab of Ornithology Cornell University Ithaca NY USA

**Keywords:** cooperation, duetting, female song, joint territory defense, suboscine

## Abstract

Duets in breeding pairs may reflect a situation of conflict, whereby an individual answers its partner's song as a form of unilateral acoustic mate guarding or, alternatively, it may reflect cooperation, when individuals share in territory defense or safeguard the partnership. The degree of coordination between the sexes when responding to solo versus paired intruders may elucidate the function of songs in duets. We examined this issue in a study with rufous horneros (*Furnarius rufus*), a duetting, socially monogamous Neotropical species with low levels of extrapair paternity. We exposed social pairs during the nonbreeding season to playbacks of duets, male solos, female solos, and control heterospecific songs. Partners approached all conspecific stimuli together and responded by singing quickly, at higher rates and by coordinating ~80% of their songs into duets. For both sexes, most response variables (seven of nine) did not vary across conspecific treatments. These results suggest that partners duet and coordinate behaviors to cooperatively defend common territories. However, females spent more time in territorial vigilance, and partners were highly coordinated (correlated responses) in response to duets and female solos in comparison with male solos. This indicates that female intrusions (paired or solo) might be more threatening than male intrusions in the nonbreeding season, especially for territorial females, and that females are less cooperative with their partners in territory defense against male intruders.

## Graphical abstract

We simulated solo and paired territorial intrusions to rufous hornero partners. Partners coordinate songs and behaviors to cooperatively defend their territories. However, partners were highly coordinated against female (solo or paired) than male intrusions derived from a weaker female response to male intrusions. We suggest that females are least cooperative with their partners when defending territories against other males.

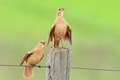

## 1 INTRODUCTION

Duets are coordinated vocal displays normally performed by breeding partners (Farabaugh, 1982). Duetting may be driven by cooperation (Hall, 2009; Logue, 2005), conflict (Tobias & Seddon, 2009), or both (Grafe & Bitz, 2004) and can mediate communication between partners (Logue, 2007) or be directed at an external audience (neighbors, strangers) (Hall, 2004). Although several nonmutually exclusive hypotheses have been proposed to explain the function of duets (Hall, 2004), two ideas have received the most attention (Dahlin & Benedict, 2013; Hall, 2009): The joint territory defense hypothesis (Robinson, 1949) and hypotheses associated with acoustic mate guarding (Rogers, Langmore, & Mulder, 2006; Seddon & Tobias, 2006).

The joint territory defense hypothesis proposes that partners duet cooperatively to establish, maintain, or defend common resources or territories (Bradley & Mennill, 2009; Seddon & Tobias, 2003). In this case, duets represent a stronger territorial signal than do solo songs, by transmitting information about numeric advantage or as a quality signal arising from song synchronization (Hall & Magrath, 2007; Kovach, Hall, Vehrencamp, & Mennill, 2014). The mate guarding hypotheses, on the other hand, suggest that duets may arise from conflict or cooperation between the mated partners. Mate guarding based on sexual conflict occurs when an individual answers its partner's song in an attempt to acoustically mate guard, for example, to intimidate rivals or discourage the partner from pursuing extrapair mates (Kahn, Moser‐Purdy, & Mennill, 2018; Rogers et al., 2006; Tobias & Seddon, 2009). Alternatively, mate guarding can also occur if divorce is costly for both partners (Choudhury, 1995), and they have a common interest in maintaining the pair bond (Griggio & Hoi, 2011; van den Heuvel, Cherry, & Klump, 2014). In this latter case (the “mutual mate guarding hypothesis”), duets are cooperative and are used to safeguard the pair bond itself (Grafe & Bitz, 2004; Hall, 2009; van den Heuvel et al., 2014; Sonnenschein & Reyer, 2010).

Researchers have tested these hypotheses through playback experiments, comparing individual aggressive responses toward simulated individual (solos) versus paired (duets) intruders (Douglas & Mennill, 2010) (Table 1). If duet functions in defense of a joint territory, one would expect a stronger and more highly coordinated response of residents toward playbacks of duets than to playbacks of solos (Douglas & Mennill, 2010; Odom & Omland, 2018), or at the very least, an equivalently aggressive response to playbacks of duets and solos (Benedict, 2010). Territory defense may be sex‐specific (Hall, 2009), when opposite‐sex intrusions are less threatening than same‐sex or pair intrusions, and duetting facilitates partner division of labor in territory defense (Templeton, Rivera‐Cáceres, Mann, & Slater, 2011). In contrast, if duet functions to guard a mate, one would predict a stronger, albeit poorly coordinated, acoustical and physical response toward same‐sex solos and a weaker response toward opposite‐sex solos (Rogers et al., 2006; Seddon & Tobias, 2006). Finally, if duet functions in mutual mate guarding, a stronger and highly coordinated response toward male and female solos versus duets would be expected, assuming that solos are a greater threat to the partnership than are duets (Templeton et al., 2011).

**Table 1:** Predicted response to playbacks of solos and duets according to the main hypotheses for song function in duets (modified from van den Heuvel et al., 2013)

Hypotheses	Territory defense		Mate guarding		
	Joint	Sex‐specific	Unilateral	Avoid mate replacement/injury	Mutual
Strongest response to	Duet/conspecific songs	Duet or duet & same‐sex solo	Same‐sex solo	Opposite‐sex solo	Solos
Weakest response to	Solos or none	Opposite‐sex solo	Opposite‐sex solo	Same‐sex solo	Duet
Response coordination	High (duet/conspecific songs)	High (duet)			High (solos)

Comparative and empirical studies provide strong support for the joint territory defense hypothesis (Dahlin & Benedict, 2013; Hall, 2009; Tobias et al., 2016). However, some studies indicate that duets may have multiple adaptive functions (Benedict, 2010; Dingle & Slabbekoorn, 2018; Grafe & Bitz, 2004). Indeed, Dahlin and Benedict (2013) estimate that 65% of studied species have multifunctional duets, of which more than 20% are both cooperative and conflict‐based. For instance, in red‐backed fairy‐wrens (*Malurus melanocephalus*), seasonal patterns of duetting and stronger responses to playbacks of duets than solos support the joint territory defense hypothesis (Dowling & Webster, 2013, 2016), but unattractive (younger, brown) males in this species also answer partner songs to acoustically guard paternity (Dowling & Webster, 2017). Few studies have focused on the coordination between partners in response to territorial intruders, albeit it may strongly contribute toward understanding the function of coordinated songs (Benedict, 2010; Dahlin & Wright, 2007; Hall & Peters, 2008; Quirós‐Guerrero, Janeiro, Lopez‐Morales, Cresswell, & Templeton, 2017).

We studied the degree to which partners coordinate aggressive response to the playback of solos versus duets in the rufous hornero (*Furnarius rufus*): a socially monogamous, duetting Neotropical bird with low extrapair paternity (3% of offspring; Diniz, Macedo, & Webster, 2019). Previous studies found evidence that territorial intrusions are common (mean = 0.7/h) and partners coordinate approximately 60% of their songs into duets and sing to defend common territories and their partnership (Diniz, Silva‐Jr, Webster, & Macedo, 2018), and that duet traits are related to territorial quality and reproductive success (Diniz et al., 2019). These studies indicate a high degree of cooperation between partners in this species, making it an ideal model to investigate the link between duet and behavioral coordination between partners.

Here, we investigate key predictions for duet function relative to the territory defense and mate guarding hypotheses (Table 1). Specifically, we address two questions linked to these predictions: (a) Do the sexes coordinate their aggressive and vocal responses to playbacks of solos versus duets? (b) Do the sexes respond differently to the simulated intrusion of solos versus duets? To answer these questions, we broadcast four treatments (i.e., duet, female solo, male solo, and a heterospecific control) to each mated pair during the nonbreeding season, and scored behavioral and vocal responses, as well as the coordination between partners in playback response. This experiment allows us to explore whether duets serve multiple functions simultaneously.

## 2 MATERIALS AND METHODS

### 2.1 Study subjects and area

The rufous hornero (Furnariidae) is a ground foraging species inhabiting disturbed open habitats across central and southern South America (Remsen & Bonan, 2016; Sick, 2001). They live in year‐round territories and breed seasonally in domed nests (Diniz, Silva‐Jr, et al., 2018; Fraga, 1980; Shibuya, Braga, & Roper, 2015). Both parents contribute equally to parental care (Massoni, Reboreda, López, & Aldatz, 2012). Juveniles delay dispersal, staying in their parents' territories from four to nine months (Diniz, Silva‐Jr, et al., 2018; Fraga, 1980).

**Figure 1:**
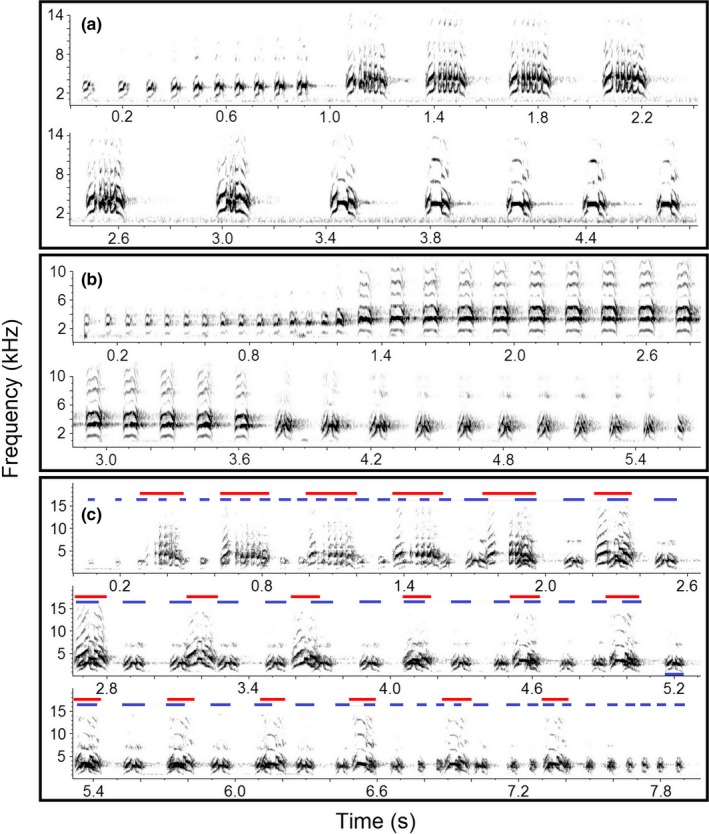
Sound spectrograms of female solo (a), male solo (b), and duet (c, female phrase in red, male phrase in blue) in the rufous hornero. Adapted from Diniz, Silva‐Jr, et al. (2018), Diniz et al. (2019)

These birds often sing two sex‐specific song types that can be coordinated in polyphonal duets (Amador, Trevisan, & Mindlin, 2005; Laje & Mindlin, 2003; Roper, 2005) and a few, slight variations of these song types that can also be coordinated into duets (Figure 1; P. Diniz, unpublished data). These song variations consist in replacement and/or omission of a syllable type, but apparently keeping the general rhythm and structure. Solo songs and duet contributions have similar structures, but differ in duration (solos are shorter) and rhythm (P. Diniz, unpublished data, Laje & Mindlin, 2003). Duets occur more often than expected by chance (P. Diniz, unpublished data).

We studied 16 territorial social units (10 pairs with juveniles and 6 pairs without juveniles) of the rufous hornero from an urban, partially banded population on the campus of the University of Brasilia, Brasilia, Brazil (15°45′S, 47°51′W) that has been monitored since 2013. The playback experiment in the field ran from January to April 2014, which corresponds to the first half of the nonbreeding season (Fraga, 1980; Shibuya et al., 2015). Sexual conflict is presumably less intense in the nonbreeding season than in the breeding season, which makes this an adequate season to study the coordination between partners in territory defense (e.g., Hall & Peters, 2008). Moreover, territorial interactions (number and duration) do not vary between breeding and nonbreeding seasons for both sexes in the rufous hornero (Diniz, Silva‐Jr, et al., 2018). Studied adults were molecularly sexed (*n* = 30) or had their sexes assigned based on their partner's sex (*n* = 2). Banding and trapping procedures are described in Diniz, Ribeiro, Rech, and Macedo (2016) and occurred during the breeding season that preceded this experimental procedure.

The 16 focal social units contained from two to five individuals when the experiment started (mean ± *SD *= 3.06 ± 1.06), and only four juveniles, from two social units, were banded. All studied pairs had one breeding attempt in the previous breeding season (from August to December 2014) (P. Diniz, unpublished data). We are confident that all unbanded juveniles hatched in the previous breeding season due to their distinctive juvenile morphology (black and short bill, slender body, and light plumage coloration; Fraga, 1980; Diniz, pers. obs.). Social unit size remained stable during the experiment, except for three units, in which juveniles were absent during part of the playback trials, probably due to short‐term movements across territories.

### 2.2 Playback stimuli

The experiment consisted of playing back four different stimuli (heterospecific control, male solo, female solo, and duet) to each studied social unit using a single‐speaker playback design. The rufous hornero has longer intersong intervals, emitting only 5.38 ± 3.52 songs/h (mean ± *SD*, *n* = 161 trials; P. Diniz, unpublished data). Thus, we used playbacks of only one song per treatment per social unit. We recorded nonplayback induced songs of sexed adults from the studied population to make the conspecific playback stimuli. These recordings were made with a Marantz PMD660 recorder (settings: WAVE, 48 kHz sampling rate, 24‐bits accuracy) and a Sennheiser ME66/K6 microphone up to 6 hr after sunrise, from August 2013 to January 2014. We recorded 59 solos from 23 adults in 15 social units and 137 duets from 18 social units.

We selected 15 high‐quality recordings (five for each conspecific treatment) to make the conspecific stimuli. We made sure each stimulus did not come from neighbors (<500 m or <5 consecutive territories) to avoid neighbor‐stranger effects on playback response (Radford, 2005; Wiley, 2013). We used songs from a syntopic duetting species, the great kiskadee (*Pitangus sulphuratus*), as a heterospecific control in our playback experiment. Both species are suboscines, abundant (Jebai et al., 2009), and sedentary in our study area. These species forage on different strata and probably do not compete for resources (Munin, Fischer, & Longo, 2012). We recorded four high quality great kiskadee songs in total (1 solo and 3 duets) from birds of noncontiguous territories (>200 m apart) in our study site and used an additional duet recorded in a nearby area (27 km from the study site, recording: Song Meter SM2, settings: WAVE format, sampling rate = 44.1 kHz, 16‐bits accuracy).

We created each playback stimulus in three steps using Raven Pro 1.5 and Audacity: (a) filtering low‐frequency (<500 Hz), (b) normalizing the maximum amplitude of each signal (−1.0 dB), and (c) adding a silent period of 10 s before and after each signal. We stored the stimuli in WAVE 16‐bits accuracy. Mean signal duration was 5.55 ± 2.01 s (*SD*) across playback stimulus (range = 2.20–9.55 s). Duet length is normally longer than solo length in the rufous hornero (P. Diniz, unpublished data), and this is reflected in the duration of our playback stimuli (mean ± *SD*, duets: 7.85 ± 1.13 s, female solos: 4.04 ± 1.49 s, male solos: 4.00 ± 0.78 s, and control: 6.30 ± 1.38 s). If the stimuli duration itself impacts playback response, we would expect differences in the response to duets (longest song stimuli) compared with solos, and no differences in the strength of playback response between female solos and male solos. These were not the observed patterns (see Section 3). The stimulus identity was assigned at random but avoiding those recordings that came from neighbors. Overall, we repeated the same stimulus 2–4 times (mean ± *SD *= 3.2 ± 0.77 playback trials/stimuli) and analyzed data with mixed models (see Section 2.5) to avoid pseudoreplication of playback stimuli (McGregor, 2000).

### 2.3 Playback experiment

We played back the four stimuli (heterospecific control, male solo, female solo, and duet) to each studied social unit in nonconsecutive days (mean ± *SD *= 4.31 ± 3.36 day‐intervals; *n* = 52 intervals) to prevent habituation (Harris & Haskell, 2013). All playback trials were carried out 1.89 ± 1.14 hr (i.e., 08:02 ± 1:08) after sunrise (mean ± *SD*, *n* = 64 trials). We presented the broadcast of the four stimuli to each social unit in random order. There were only two out of 24 possible stimuli order combinations that were repeated in different social units.

The rufous horneros usually build their nests throughout the year, even during the “non‐breeding” season (Fraga, 1980). To broadcast each stimulus, we placed one speaker (TSI Supervoz II 1210, frequency response: 80 Hz–12 kHz) on the nest substrate (i.e., tree or light pole), given that in nature intruders may sing on the nest substrate of territorial birds (Diniz, pers. obs.). Moreover, we wanted to make sure birds would hear the broadcast. Our rufous hornero population lives in a noisy environment, and partners defend small territories (0.7 ha, Diniz et al., 2019) and seem to spend most of their time near the nest substrate (Diniz, pers. obs.).

We did not use stereo or dual duet playback (Douglas & Mennill, 2010), because males and females overlap phrases in duets in the same frequency range (Figure 1), thus we could not extract male and female song contributions (Hall & Peters, 2008). In addition, single and stereo speaker playback may elicit similar playback responses in rufous horneros, because males and females normally coordinate songs when they are very close to each other (median distance = 0.76 m, *n* = 22 social units and 138 duets plus choruses, P. Diniz, unpublished data).

We attached the speaker to a metal rod at an approximate height of 5 m, which corresponds to the average height that rufous horneros sing in our population (P. Diniz, unpublished data). We positioned the speaker parallel to and facing the ground (birds forage on the ground) and attached the metal rod to the nest substrate. Rufous horneros sing duets at approximately 92 dB maximum amplitude (estimated for 1 m distance from the bird) as measured by a sound level meter (model SEW 2310SL) at 20.99 ± 7.96 m from the birds in the field (91.82 ± 2.63 dB, *n* = 10 pairs; P. Diniz, unpublished data; distance effects on amplitude corrected according to van den Heuvel, Cherry, & Klump, 2013). Therefore, we calibrated the speaker volume in silent conditions in the laboratory to broadcast the stimulus at 92 dB maximum sound level at 1 m from the speaker. Finally, we connected the speaker to a cell phone with a 30 m cable and triggered the stimulus with a WAVE player application (Rocket Player) when both focal adults were within 60 m of the speaker. The birds were easily spotted in the open, urban habitat, though not vocalizing constantly.

After broadcasting each playback stimulus, one or more observers (mean ± *SD *= 2.73 ± 0.60, range = 1–4, *n* = 64 trials) recorded adult behavior and their songs during 15 min (recording apparatus: Marantz PMD660 recorder, Sennheiser ME66/K6 microphone). We were able to track the birds for 92 ± 18% of each focal period (mean ± *SD*; *n* = 128 bird trackings). After finishing each trial, we used a measuring tape to estimate the spatial position and movement of birds, which occurred in response to the playback. It was not possible to record data blind because our study involved focal animals in the field.

### 2.4 Playback response

Rufous horneros normally respond to conspecific playbacks by approaching and perching high on the speaker substrate (tree or light pole) instead of approaching the speaker itself or branches close to it, which is a typical response for many birds (Dahlin & Wright, 2012; Funghi, Cardoso, & Mota, 2015; Hall, 2000; Rogers et al., 2006). Rufous horneros then usually sing once and do not sing again for an average of 5.63 ± 3.81 min (mean ± *SD*, *n* = 110) after the playback. Therefore, we chose playback response variables based on the unusual playback response of this species. Juveniles rarely initiate group singing but can join parent‐initiated songs (Diniz, Silva‐Jr, et al., 2018), and we did not have enough sample size to compare pairs with and without juveniles. Thus, we focused on the playback responses of adult birds.

Regarding bird movement responses, we measured: (a) the closest horizontal distance of the bird to the speaker after its first movement toward the playback (measured for 94/96 bird trackings); and (b) the height of each bird after the first approach to the speaker (85/96 bird trackings). We combined these variables and used the Pythagorean Theorem to estimate the real postplayback distance between each bird and the speaker (“closest approach,” hereafter).

We also estimated the time spent in “territorial vigilance” (i.e., sentinel‐like behavior), where the bird was perched, scanning or singing, relatively immobile or moving among perches in the same substrate (see Tobias & Seddon, 2000). Rufous horneros often alternate between foraging on the ground and staying in territorial vigilance perched on the top of tall trees (Diniz, pers. obs.). Birds often sang at the beginning of a territorial vigilance bout (Diniz, pers. obs.). We estimated other behavioral response variables based on the approach to the speaker and spatial movement (Table 2).

**Table 2:** Measurements taken at individual level of behavioral and vocal responses to the playback by adult rufous horneros

Behavioral response	
Approach	Approaching the speaker or not: distance to the speaker reduced by more than 4 m during the first 30 s after the broadcast stimulus
Closest approach	Distance (m) between the bird and the speaker after the broadcast stimulus and after the bird approached the speaker
Singing location	Probability of song at the speaker tree/light post during the 15‐min playback trial
Territorial vigilance	Proportion of time spent perched in vigilance

Vocal response	
Latency to sing (s)	Latency to sing after the stimulus was broadcast
Song rate	Number of songs (solos and duet phrases) by each bird
Singing role	Song initiator or song responder
Latency to answer partner‐initiated song	Latency to sing (s) after the partner initiated a song
Song duration	Duration (s) of solos and duet contributions

We selected and quantified songs emitted by each bird and assigned the singing role (initiator or responder) for each song. We did not quantify song answering rate (Logue, 2005) since the birds emitted only 1.89 ± 1.10 songs/trial (mean ± *SD*, *n* = 122 songs, 61 trials). We classified as song initiator the bird that started a song before its partner (Hall & Peters, 2008), regardless of whether it was answered (i.e., duets or chorus) or not (i.e., solos) by its partner (Diniz, Silva‐Jr, et al., 2018). The song responder was the bird that sang after its partner had sung, thus creating a duet or chorus (Logue & Krupp, 2016).

We analyzed vocal behavior data using Raven Pro 1.5. We measured song duration for all songs at the individual level: solos and each contribution to a duet or chorus. Finally, we measured the latency to answer partner‐initiated song (Table 2).

### 2.5 Statistical analyses

We analyzed the effects of playback treatments on approach response (approach or not approach the speaker) of each sex with Fisher's exact test (pooled data from different individuals) using Past 3.14 (Hammer, Harper, & Ryan, 2001). For this analysis, we pooled data from different individuals because there was little variation among social units (see Section 3) and only a data point for each individual. We analyzed the remaining playback response data with linear mixed models (LMM) or generalized linear mixed models (GLMM) in R (Appendix S1, Table S1). We did not use principal component analyses (PCA) to reduce the number of response variables (McGregor, 1992) for two reasons: our response variables differ in sample size, and we would lose power by combining variables; and not all of our response variables are normally distributed, making them inappropriate for PCAs (Quinn & Keough, 2002).

As predictor factors, we included main effect of playback treatment in all the models and the main effects and interaction of playback treatment and sex in all models at the individual level. We included the social unit type (pairs with juveniles or pairs without juveniles) and order of the playback stimulus as covariates in all models. All continuous variables were scaled to obtain comparable *β* estimates (Zuur, Hilbe, & Ieno, 2013). We added random factors, such as stimulus identity, social unit, and individual identities to avoid pseudoreplication, and additional predictors to model specific response variables (Table S1). To elucidate the interactions effects, we also built models separately for each sex, keeping the other terms listed above and not including social unit identity as a random term.

We also analyzed how playback treatments influenced the coordination between partners in their response to playback (closest approach, territorial vigilance, song rate, and phrase duration). We used the same modeling approach described above (except for territorial vigilance), but with female playback response as the response variable and the correspondent male playback response as fixed effect for each treatment level (Table S7). We did not build a model containing the interaction between partners' response and treatment because it resulted in a high value of variance inflation factor (VIF). The structure of random terms was changed accordingly. For modeling coordination, we used the arc‐sine transformation for proportion of time spent in territorial vigilance.

We applied backward stepwise model selection to choose the top‐fitted model. We verified the significance of predictors with likelihood‐ratio tests (LRT), keeping the random terms in all models (Zuur, Ieno, Walker, Saveliev, & Smith, 2009). Once we found a significant result in the top‐fitted model, we applied post hoc tests using the packages “lsmeans” and “multcomp” (Hothorn, Bretz, & Westfall, 2008; Lenth, 2015) to generate estimates for between‐levels differences while controlling for multiple tests (false discovery rates).

To model time spent in territorial vigilance, we did not consider the playback trials where the bird was absent for more than 50% of the time (*n* = 6 out of 128 cases, 4.69%). To model latency to sing, we did not correct for the distance between the bird's positions before and after the playback, because there was no relationship between these variables in a premodeling scenario (*χ* ^2^ = 0.86, *p* = .35). Outliers were identified by box plot inspections (Zuur et al., 2009) and removed before analyzing variation in song duration (*n* = 7) and in the correlation analysis in song duration between partners (*n* = 1). Model selection and detailed sample sizes steps are described in the Appendix S1.

## 3 RESULTS

### 3.1 Coordination between partners

**Figure 2:**
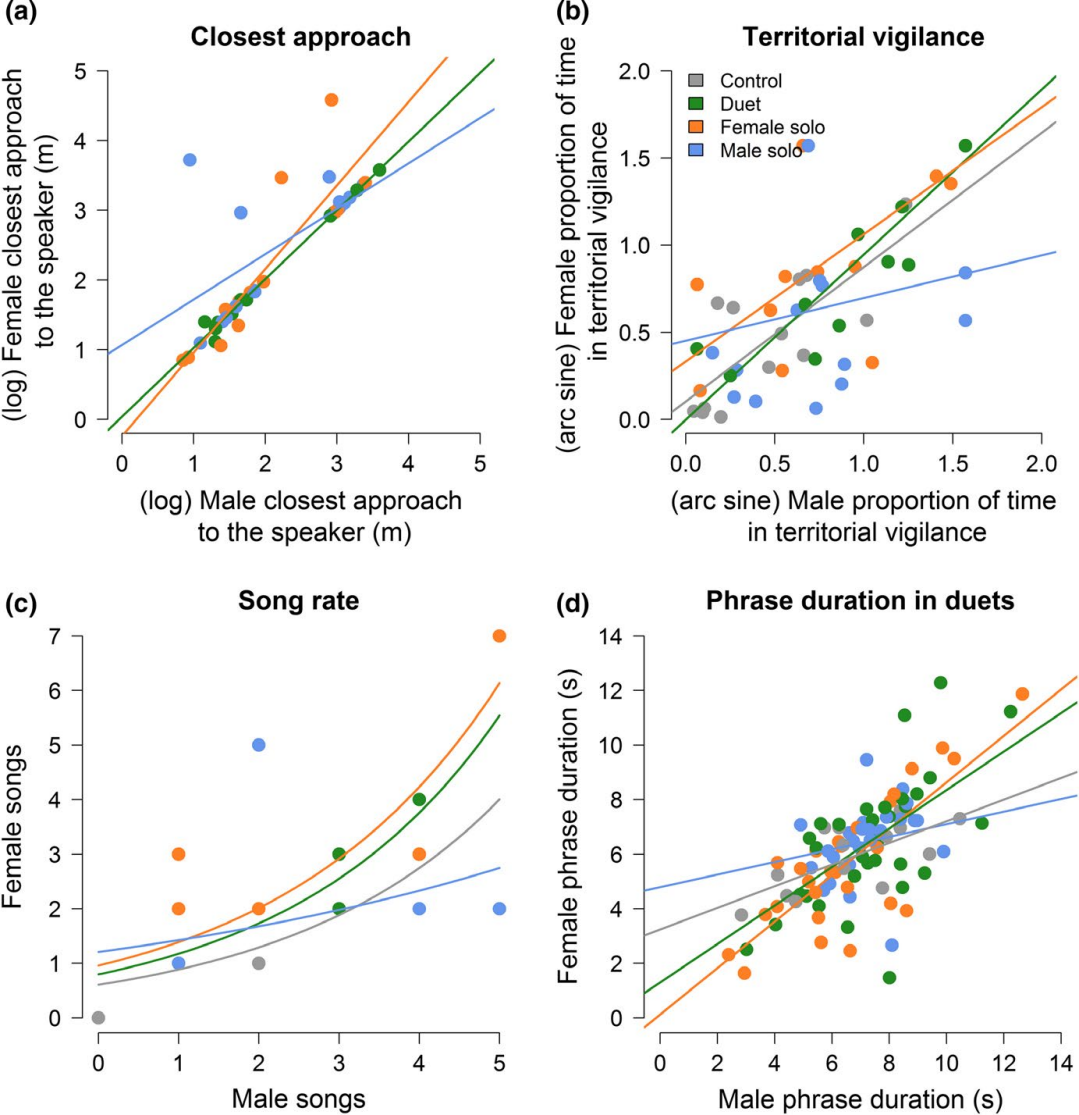
Variation in the correlation between partners in playback responses (closest approach to speaker, time spent in territorial vigilance, song rate, and phrase duration in duets). Because birds did not approach the heterospecific playback, correlation in closest approaches was only compared among conspecific treatments. Lines represent model coefficients

**Figure 3:**
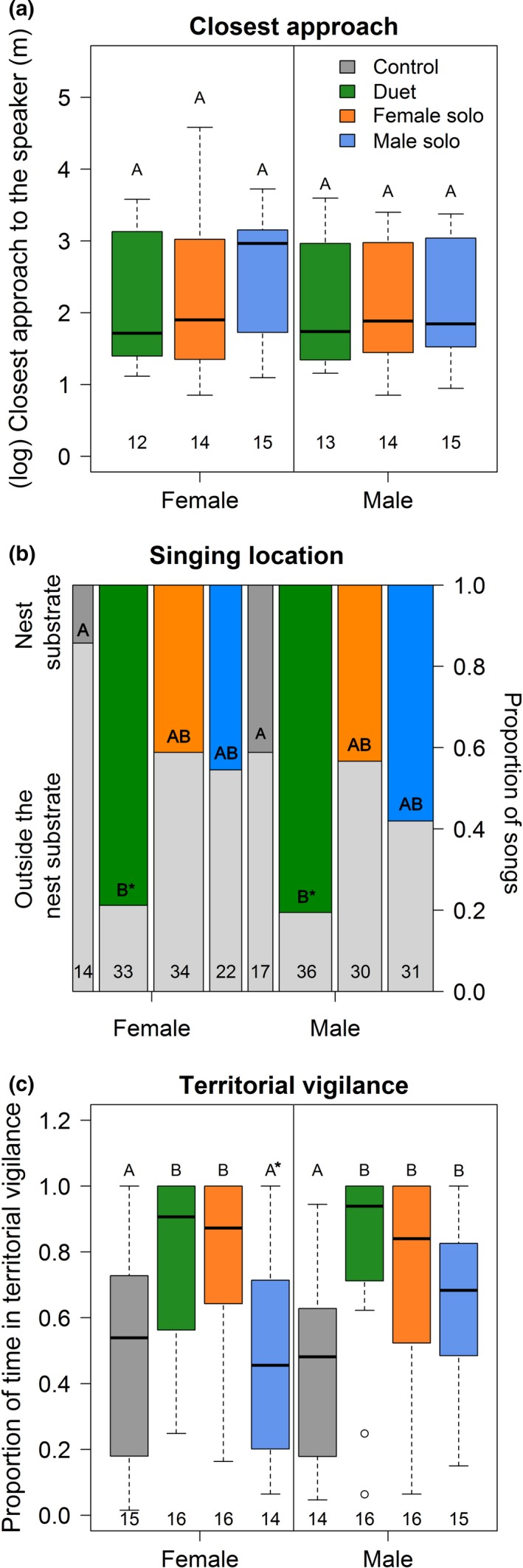
Box plots (panels a and c) and bar plot (panel b) showing behavioral responses of females and males to playbacks of female solo, male solo, duet, and an heterospecific control. Different letters show statistical differences between factor levels. Sample sizes are shown at the bottom of the boxes (panels a and c: number of trials; panel b: number of songs). For panel b, dark gray bar and colored bars indicated proportion of songs given at the nest substrate. For all panels, post hoc comparisons were made between treatment levels (sexes analyzed separately and combined), because sex did not affect these behavioral responses. Closest approach does not include control treatment when no bird approached the speaker. Significant differences only existed when sexes were combined (panel b) or separated (panel c) for analyzes

Rufous hornero partners coordinated all their behavioral responses to the playback types. Females and males typically approached the speaker in response to nearly all conspecific playbacks (females in 98% and males in 96% of the 48 trials; Fisher's exact test, both sexes, *p* < .0001) and did not approach the heterospecific control playback. Female and male closest approaches to the speaker were positively correlated within pairs (*r_p_ *= .82, *p* < .0001, *n* = 41 trials; Figure 2a) and did not vary between sexes (LMM: sex: likelihood‐ratio test [LRT] = 2.94, degrees of freedom [*df*] = 1, *p* = .09, *n* = 48 trials; mean ± *SD* for female: 15.82 ± 13.45 m; and male: 11.98 ± 8.37 m, *n* = 16 social units; Figure 3a). In addition, singing location, that is, the probability of singing at the nest substrate did not vary between sexes (GLMM: sex: LRT = 1.95, *df* = 1, *p* = .16, *n* = 59 trials; Figure 3b). Finally, females and males spent an equal amount of time in territorial vigilance in response to the playback types (LMM: sex: LRT = 0.05, *df* = 1, *p* = .83, *n* = 63 trials; mean ± *SD* for female: 8.11 ± 3.34 min, *n* = 15 females; and male: 9.08 ± 3.12 min, *n* = 16 males; Figure 3c), and the time spent in territorial vigilance was positively correlated between partners when combining data from all treatments (*r_p_* = .79, *p* < .0001, *n* = 59 trials).

**Figure 4:**
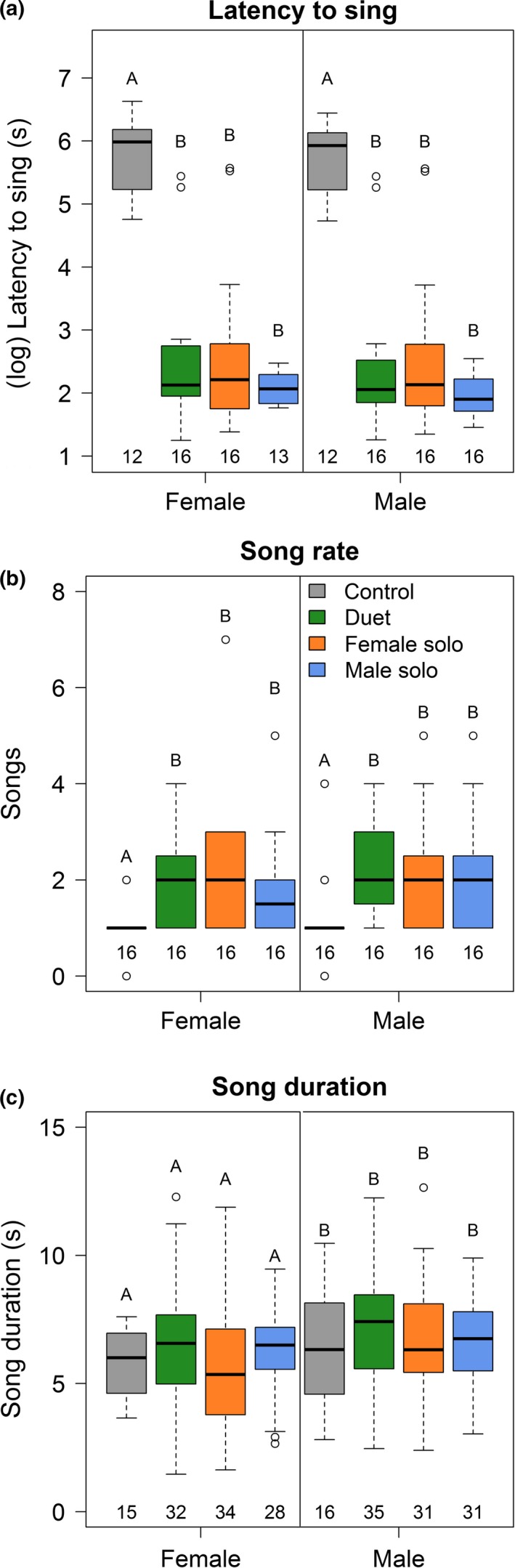
Box plots showing vocal responses of females and males to playbacks of female solo, male solo, duet, and an heterospecific control. Different letters show statistical differences between factor levels. Sample sizes are shown at the bottom of the boxes (panels a and b: number of trials; panel c: number of songs). For panels a and b, post hoc comparisons were made between treatment levels (sexes combined), because sex and the interaction between sex and treatment did not affect these behavioral responses. For panel c, post hoc comparisons were made between sexes (treatment levels combined), because treatment and the interaction between sex and treatment did not affect these vocal responses. Results did not differ when sexes were analyzed separately

Partners also coordinated their vocal behaviors in response to the playback. Both sexes often sang after approaching the speaker (females in 90% and males in 92% of 48 trials) and coordinated most of their songs into duets (78%, *n* = 130 songs). In addition, both the latency to sing (LMM: sex: LRT = 0.71, *df* = 1, *p* = .40, *n* = 61 trials; Figure 4a) and song rate did not vary between the sexes (GLMM: sex: LRT = 0.21, *df* = 1, *p* = .65, *n* = 64 trials; Figure 4b). Finally, song rate and phrase duration in duets were positively correlated between partners when combining data from all treatments (song rate: *r_p_* = .72, *p* < .0001, *n* = 64 trials; song duration: see below; Figure 2c,d).

Despite these similarities, responses to the playback varied between the sexes in some ways irrespective of playback type. The probability of initiating a song was higher for males than for females (GLMM: sex: LRT = 7.35, *df* = 1, *p* = .007, *n* = 61 trials; *β* = 1.12, 95% CI = 0.3–1.9), such that most (62.75%, *n* = 102 duets) duets were the result of females responding to male songs. In addition, males answered partner‐initiated songs more quickly than did females (LMM: sex: LRT = 5.35, *df* = 1, *p* = .02, *n* = 61 trials, 101 songs; mean ± *SD* for female: 1.35 ± 1.45 s, *n* = 63 songs of 16 females; and male: 0.85 ± 0.95 s, *n* = 38 songs of 14 males). Males also sang longer songs than did females (LMM: sex: LRT = 6.98, *df* = 1, *p* = .008, *n* = 61 trials, 121 songs; *β* = 0.59, 95% CI = 0.2–1.0; Figure 4c).

### 3.2 Responses to the playback types

In general, partners responded similarly to all three conspecific treatments. Both sexes started to sing quickly (LMM: treatment: LRT = 49.56, *df* = 1, *p* < .0001, *n* = 61 trials; Figure 4a) and sang at higher rates (GLMM: treatment: LRT = 13.74, *df* = 3, *p* = .003, *n* = 64 trials; Figure 4b) in response to all conspecific playbacks compared with heterospecific control (Table S5). The singing role (song initiator vs. song responder), latency to answer partner‐initiated song and song duration did not differ between conspecific playbacks and heterospecific control for either sex (Table S2).

Birds responded most strongly to duets and female solos in the contexts of territorial vigilance and coordinated response between partners. First, birds spent more time in territorial vigilance (LMM: treatment: LRT = 15.95, *df* = 3, *p* = .001, *n* = 63 trials) in response to playbacks of duets and female solos compared with heterospecific control, and females spent more time in territorial vigilance in response to female solos and duets compared with male solos when sexes were analyzed separately (Figure 3c). Males spent similar time in territorial vigilance after the three conspecific playbacks (Figure 3c). Second, partners presented a higher level of correlated responses to playbacks of duets and female solos compared with male solos (Figure 2, Table S7). Correlation between the sexes in closest approach to speaker (*r_p_* = 1.00, *p* < .0001, *n* = 12 pairs) and time spent in territorial vigilance (*r_p_* = .93, *p* < .0001, *n* = 16 pairs) peaked in response to duets, whereas the correlation between the sexes in song rate (*r_p_* = .85, *p* < .0001, *n* = 16 pairs) and song duration (*r_p_* = .81, *p* < .0001, *n* = 16 pairs) was strongest in response to female solos.

Both sexes sang more often at the nest substrate in response to duet playbacks compared with playbacks of heterospecific controls (GLMM: treatment: LRT = 18.34, *df* = 3, *p* = .004, *n* = 59 trials; Figure 3b; Table S5), although these results were not significant when the sexes were analyzed separately (female: *p* = .07, male: *p* = .09). The probability of singing at the nest substrate did not vary across conspecific treatments (Figure 3b; Table S5). Territorial vigilance (*r_p_* = .49, *p* = .07, *n* = 14 pairs), song rate (*r_p_* = .37, *p* = .15, *n* = 16 pairs) and song duration (*r_p_* = .30, *p* = .15, *n* = 16 pairs) were not correlated between the sexes when responding to male solos (Figure 2).

## 4 DISCUSSION

### 4.1 Coordination between partners

Rufous hornero partners converge remarkably in their playback responses, such that both sexes typically approach the speaker and duet in response to the majority of conspecific playbacks. Playbacks of conspecific songs induced an equivalently aggressive response of territorial females and males in seven of nine individual‐level categories of responses evaluated (Table 2, Figure 2). The probability of initiating a song or answering a partner's song did not differ among playback treatments. We also found a strong correlation between the sexes in several physical and vocal behavioral traits, especially during aggressive contexts (conspecific playbacks), but also during the nonaggressive (control playback) context. Taken together, these results suggest that partners coordinate aggression directed toward intruders (Hall & Peters, 2008; Quirós‐Guerrero et al., 2017).

A high degree of convergence and coordination between the sexes in playback responses has been found for a few other species (Benedict, 2010; Dahlin & Wright, 2007; Hall & Peters, 2008; Quirós‐Guerrero et al., 2017). In yellow‐naped amazons (*Amazona auropalliata*), partners did not differ in the approach behavior or vocal output (Dahlin & Wright, 2012). Similarly, in purple‐crowned fairy‐wrens (*Malurus coronatus*), partners coordinate their approach to the speaker and their vocal output in response to playbacks of duets (Hall & Peters, 2008). However, the majority of duetting birds studied previously show some sort of sex‐specificity in playback responses (van den Heuvel et al., 2014; Levin, 1996; Rogers et al., 2006; Seddon & Tobias, 2006) or limited coordination during territory interactions with strangers (Hall, Rittenbach, & Vehrencamp, 2015; Hathcock & Benedict, 2018). Female canyon wrens (*Catherpes mexicanus*), for instance, are more likely to sing but not to duet in response to conspecific challenges (Hathcock & Benedict, 2018). Thus, convergence in response to playbacks is not the usual pattern and may indicate the strength of cooperation between partners (Hall & Peters, 2008).

Rufous hornero males initiated most songs, answered partner‐initiated songs more promptly and sang longer phrases in duets than did females in both aggressive and nonaggressive contexts (Diniz, Silva‐Jr, et al., 2018). This suggests that males have a primary role in territory defense (Brumm & Goymann, 2018). This is particularly interesting because in addition to the cooperative and strongly united responses of rufous hornero partners to all conspecific stimuli, sexual selection may still play a role in song evolution in this species. Male‐biased singing effort and answering rates are common among both duetting and nonduetting bird species (Catchpole & Slater, 2008; Hall, 2009) and deserves further investigation.

Further research should investigate if the high coordination between partners for territory defense is consistent throughout the year, especially during the breeding season.

### 4.2 Responses to the playback types

We evaluated predictions for duetting functional hypotheses of territory defense, mate guarding, and their variations (Table 1). Partners approached quickly and sang at higher rates in response to conspecific songs compared with the heterospecific control. For both sexes, most response variables did not vary across conspecific playback treatments. Importantly, both sexes answered partner‐initiated songs regardless of playback treatment. These results indicate that both sexes respond to conspecific intruders as similar threats, supporting the joint territory defense for duetting function (Table 1) (Dowling & Webster, 2016; Hall, 2004, 2009; Tobias et al., 2016). The joint territory defense hypothesis was also supported by previous studies in rufous horneros. These studies found that both sexes increase song rates during natural territorial intrusions by conspecifics (Diniz, Silva‐Jr, et al., 2018); female song output correlates with territory quality, and parents' duet duration correlates with postfledging survival (Diniz et al., 2019).

Females spent more time than males in territorial vigilance in the presence of a simulated female intruder (paired or not). This result supports the sex‐specific territory defense for the function of female songs in duets (Table 1) and agrees with the idea that female–female competition is an important factor driving the evolution of female signaling traits in general (Cain & Ketterson, 2013; Clutton‐brock, 2009; Diniz, Oliveira, Marini, & Duca, 2018; Rosvall, 2011). Extrapair paternity is rare (3% of offspring) (Diniz et al., 2019); divorce rate is apparently low, and there is evidence that pair bonds may last for at least 4 years in the rufous hornero (Fraga, 1980). These traits coincide with a scenario of intense intraspecific, territorial competition. A further study testing whether territorial females direct aggressions toward female rivals during simulated paired intrusions (e.g., dual‐speaker experiment) (Quirós‐Guerrero et al., 2017) may elucidate the degree of sex‐specificity in territorial defense by females.

Partners were highly coordinated when responding to paired or solo female intrusions (or strange female song), and to a lesser degree in nonaggressive contexts. In the presence of simulated male intruders (or male songs), the territorial responses of partners were weakly coordinated when compared with the nonaggressive context (Figure 2). These results could be driven by a less motivated response of territorial females against strange males or male song, suggesting that intruder males are a lower threat than intruder females to territorial females (Brumm & Goymann, 2018). Females spending less time in territorial vigilance in response to male solos may lead to an asymmetry in territorial vigilance between the sexes (e.g., male is perched in vigilance while female is foraging on the ground), which in turn may desynchronize the behavior of partners during territory defense. On the other hand, territorial partners may perceive female intrusions as more threatening than male solo intrusions and increase behavioral coordination to defend common territories against paired and solo female intruders.

Both partners tended to approach closest to the nest substrate in response to paired intrusions (or duet songs). Rufous hornero partners build heavy mud domed nests throughout the year (Shibuya et al., 2015), which may require a high level of nest building effort. Our results suggest that territorial partners treat paired intrusions or duets as a strong threat, as they may signal the potential loss of a nest or nest site.

In conclusion, we found remarkable cohesion and coordination between partners in playback responses and conspecific songs, indicating that pairs cooperatively duet to defend common territories during the nonbreeding season. However, we found evidence that partners paid most attention and exhibited a higher coordination in territory defense in the presence of female intruders (paired or not), and females were less vigilant against male solo intruders. We suggest that female intrusions are more threatening than male intrusions, especially for territorial females, and that females cooperate less with their partners in territory defense in the context of male solo intruders.

## CONFLICT OF INTEREST

The authors declare that they have no conflicts of interest.

## ETHICAL APPROVAL

Banding and trapping procedures were conducted as quickly as possible, no bird abandoned its nest or territory after banding procedures, and normally resumed foraging or incubation activities within 10 min. We played back only three conspecific song stimuli to each study social unit, each song broadcasted lasted less than 10 s, and song stimuli were broadcast in nonconsecutive days. Birds often returned to normal activities (foraging, nest building) within 15 min, and no bird abandoned its territory after the experiment. Thus, we believe our playback design generated minimum disturbance to birds.

## PERMITS

All applicable institutional and/or national guidelines for the care and use of animals were followed. This study was approved by the Brazilian environmental agencies “Instituto Chico Mendes de Conservação da Biodiversidade—ICMBio” (ICMBio, licence number 40806–1) and “Centro Nacional de Pesquisa para Conservação das Aves Silvestres—CEMAVE” (licence number 3886).

## Data Availability

Most of the data supporting this study are provided as supplementary information, and the remaining data, including data sheets, are available from the Dryad Digital Repository Dryad (https://doi.org/10.5061/dryad.5tb2rbp0m).
