# Supplementary material for: Partners coordinate territorial defense against simulated intruders in a duetting ovenbird
Source: Ecol Evol. 2019 Nov 12;10(1):81–92. doi: 10.1002/ece3.5821 (PMC6972807; doi:10.1002/ece3.5821)
Supplement: Supplementary file 1 [file ECE3-10-81-s001.docx]

# Supplementary Material Appendix 1

**Table S1.** Structure of full mixed models for behavioral and vocal responses. Playback treatments: ^1^all treatments (female solo, male solo, duet, heterospecific song), ^2^only conspecific treatments.

| Response variable | Family | Predictor variables | Random effects |
| --- | --- | --- | --- |
| Closest approach (log)^2^ | Gaussian | PT × SE + UT + OR | II nested in GI + SI |
| Singing location^1^ | Binomial | PT × SE + UT + OR + VR | II nested in GI + SI + PI |
| Territorial vigilance^1^ | Binomial (proportion) | PT × SE + UT + OR | II nested in GI + SI + OL |
| Latency to sing (log)^1^ | Gaussian | PT × SE + UT + OR | II nested in GI + SI |
| Song rate^1^ | Poisson | PT × SE + UT + OR | II nested in GI + SI |
| Singing role^1^ | Binomial | PT × SE + UT + OR + VR | II nested in GI + SI + PI |
| Latency to answer partner-initiated song (log)^1^ | Gaussian | PT × SE + UT + OR + VR | II nested in GI + SI + PI |
| Song duration^1^ | Gaussian | PT × SE + UT + OR + VR + SR + MD | II nested in GI + SI + PI |
| Predictor variables: PT (playback treatment), SE (sex), UT (social unit type: pairs with juveniles or pairs without juveniles), OR (order that the playback treatment was presented), VR (order of the vocal response), MD (song mode: solo or coordinated song), SR (singing role: initiator or answered), FS (distance female-speaker), MS (distance male-speaker). Random effects: GI (group identity), II (individual identity), SI (stimulus identity), PI (playback trial identity), OL (observation-level random effect to deal with overdispersion). | | | |

**Table S2.** Predictor effects in backward stepwise model selection for variation in several playback response variables of adult rufous horneros. Predictors are shown in the descending order in which they were removed from the full model until the final model, containing only significant predictors. LRT = likelihood ratio test. df = degrees of freedom. Social unit type: pair without juveniles or pair with juveniles.

| Response variable | Predictor variables | LRT | df | p |
| --- | --- | --- | --- | --- |
| Closest approach | Playback treatment × sex | 0.71 | 2 | 0.70 |
|  | Social unit type | 0.22 | 1 | 0.64 |
|  | Playback treatment | 2.13 | 2 | 0.34 |
|  | Sex | 2.94 | 1 | 0.09 |
|  | Order of the stimulus broadcasted | 4.19 | 1 | 0.04 |
| Singing location | Order of the stimulus broadcasted | 0.20 | 1 | 0.65 |
|  | Social unit type | 0.29 | 1 | 1.12 |
|  | Playback treatment × sex | 4.24 | 1 | 0.24 |
|  | Sex | 1.95 | 1 | 0.16 |
|  | Order of the vocal response | 13.75 | 1 | 0.0002 |
|  | Playback treatment | 18.34 | 1 | 0.0004 |
| Territorial vigilance | Playback treatment × sex | 0.10 | 1 | 0.75 |
|  | Sex | 0.05 | 1 | 0.83 |
|  | Social unit type | 0.11 | 1 | 0.74 |
|  | Order of the stimulus broadcasted | 0.18 | 1 | 0.68 |
|  | Playback treatment | 15.95 | 3 | 0.001 |
| Latency to sing | Playback treatment × sex | 0.64 | 3 | 0.89 |
|  | Order of the stimulus broadcasted | 0.22 | 1 | 0.64 |
|  | Sex | 0.71 | 1 | 0.40 |
|  | Social unit type | 3.29 | 1 | 0.07 |
|  | Playback treatment | 49.56 | 1 | < 0.0001 |
| Song rate | Playback treatment × sex | 0.86 | 3 | 0.84 |
|  | Order of the stimulus broadcasted | 0.05 | 1 | 0.82 |
|  | Sex | 0.21 | 1 | 0.65 |
|  | Social unit type | 1.22 | 1 | 0.27 |
|  | Playback treatment | 13.74 | 3 | 0.003 |

| **Table S2** |  |  |  |  |
| --- | --- | --- | --- | --- |
| Response variable | Predictor variables | LRT | df | p |
| Singing role | Social unit type | 0.03 | 1 | 0.87 |
|  | Order of the stimulus broadcasted | 0.27 | 1 | 0.60 |
|  | Order of the vocal response | 1.64 | 1 | 0.20 |
|  | Playback treatment × sex | 6.25 | 3 | 0.10 |
|  | Playback treatment | 0.50 | 1 | 0.92 |
|  | Sex | 7.35 | 1 | 0.007 |
| Latency to answer partner-initiated song | Order of the stimulus broadcasted | 0.07 | 1 | 0.79 |
|  | Order of the vocal response | 0.15 | 1 | 0.70 |
|  | Social unit type | 1.15 | 1 | 0.28 |
|  | Playback treatment × sex | 4.75 | 3 | 0.19 |
|  | Playback treatment | 6.32 | 3 | 0.10 |
|  | Sex | 5.35 | 1 | 0.02 |
| Song duration | Playback treatment × sex | 0.19 | 3 | 0.98 |
|  | Order of the stimulus broadcasted | 0.06 | 1 | 0.80 |
|  | Social unit type | 2.14 | 1 | 0.14 |
|  | Playback treatment | 5.97 | 3 | 0.11 |
|  | Sex | 6.98 | 1 | 0.008 |
|  | Singing role | 15.16 | 1 | <0.0001 |
|  | Song mode | 45.80 | 1 | <0.0001 |

**Table S3.** Standard deviation (SD) for random effects in the final models.

| Response variable | Playback trial | Individual ID | Group ID | Stimuli ID |
| --- | --- | --- | --- | --- |
| Closest approach |  | 0.00 | 0.75 | 0.34 |
| Singing location | 4.56 | 0.00 | 0.00 | 3.62 |
| Territorial vigilance^1^ |  | 0.00 | 1.34 | 0.00 |
| Latency to sing |  | 0.00 | 0.79 | 0.36 |
| Song rate |  | 0.00 | 0.22 | 0.00 |
| Singing role | 0.00 | 0.75 | 0.00 | 0.00 |
| Latency to answer partner-initiated song | 0.15 | 0.00 | 0.00 | 0.00 |
| Song duration | 0.46 | 0.00 | 1.03 | 0.24 |

^1^Observation-level random effect: SD = 3.39.

**Table S4.** General sample sizes (number of rows in the data frame) for modelling each playback response variable.

| Response variable | Playback treatment (duet, female solo, male solo, control) | Social unit type (pairs with /without juveniles) | Sex (female, male) | Trials, social units, stimuli |
| --- | --- | --- | --- | --- |
| Closest approach | 25, 28, 30, no control | 32, 51 |  | 48, 16, 15 |
| Singing location | 69, 64, 53, 31 | 84, 133 | 103, 114 | 59, 16, 20 |
| Territorial vigilance | 32, 32, 29, 29 | 45, 77 | 61, 61 | 63, 16, 20 |
| Latency to sing | 32, 32, 29, 24 | 42, 75 | 57, 60 | 61, 16, 20 |
| Song rate | 32, 32, 32, 32 | 46, 82 | 64, 64 | 64, 16, 20 |
| Singing role | 68, 68, 62, 34 | 93, 139 | 112, 120 | 61, 16, 20 |
| Latency to answer partner-initiated song | 32, 29, 25, 15 | 37, 64 | 63, 38 | 61, 16, 20 |
| Song duration | 67, 65, 59, 31 | 85, 137 | 109, 113 | 61, 16, 20 |

**Table S5.** Post hoc comparisons among levels of significant predictors or interactions between predictors retained in the final models. We show only results for predictors of interest and not covariates. We show estimates plus confidence intervals (5% CI and 95% CI). Estimates are only shown for significant differences (*, p < 0.05) between levels of predictors or interactions after controlling for false discovery rates. We also indicated significant results when sexes were analyzed separately (^F^ = female, ^M^ = male).

| Response variable | Predictor variables | Predictor levels | Estimate (CI: 5%, 95%) |
| --- | --- | --- | --- |
| Singing location | Playback treatment | Control – duet* | –10.45 (–17.6, –3.3) |
| Territorial vigilance | Playback treatment | Control – duet*^FM^ | –3.98 (–5.8, –2.2) |
|  |  | Control – female solo*^FM^ | –3.66 (–5.4, –1.9) |
|  |  | Duet – male solo*^F^ | 2.57 (0.8, 4.4) |
|  |  | Female solo – male solo*^F^ | 2.25 (0.5, 4.0) |
| Latency to sing | Playback treatment | Control – duet*^FM^ | 3.23 (2.7, 3.8) |
|  |  | Control – female solo*^FM^ | 3.10 (2.5, 3.7) |
|  |  | Control – male solo*^FM^ | 3.62 (3.0, 4.2) |
| Song rate | Playback treatment | Control – duet*^FM^ | –0.68 (–1.1, –0.3) |
|  |  | Control – female solo*^FM^ | –0.69 (–1.1, –0.3) |
|  |  | Control – male solo*^FM^ | –0.60 (–1.0, –0.2) |
| Singing role¹ | Sex | Female – male* | 1.12 (0.3, 1.9) |
| Latency to answer partner-initiated song | Sex | Female – male* | 0.53 (0.1, 1.0) |
| Song duration | Sex | Female – male* | –0.59 (–1.0, –0.2) |

¹ Probability of being the song responder instead of the song initiator

**Table S6.** Beta (β) estimates for covariates retained in the top models for each response variable. The estimates for the main predictors can be found in the main text.

| Response variable | Predictor variables | *β* ± SE | *z* ratio or *t* value |
| --- | --- | --- | --- |
| Closest approach | Order of the stimulus broadcast | –0.16 ± 0.07 | –2.20 |
| Singing location | Order of the vocal response | –1.19 ± 0.39 | –3.03 |
| Song duration | Singing role: initiator – answerer | 0.91 ± 0.23 | 3.95 |
|  | Song mode: coordinated song – solo | 3.47 ± 0.49 | 7.14 |

**Table S7.** Correlation between female and male responses to each playback treatment. We modelled female response in function of partner’s response for each playback treatment. We show results of likelihood ratio tests (LRT) on GLMM or LMM models and Pearson product-moment correlation between female and male responses.

| Response variable | Predictor variable | Treatment | LRT (df = 1) | p | Estimate ± SE | r_p_ (df) | p | n |
| --- | --- | --- | --- | --- | --- | --- | --- | --- |
| Female closest approach (log)^1^ | Male closest approach (log) | Duet | 51.09 | < 0.0001 | 0.99 ± 0.03 | 1.00 (10) | < 0.0001 | 12 pairs |
|  |  | Female solo | 21.42 | < 0.0001 | 1.20 ± 0.18 | 0.89 (12) | < 0.0001 | 14 pairs |
|  |  | Male solo | 7.07 | 0.008 | 0.65 ± 0.23 | 0.61 (13) | 0.015 | 15 pairs |
| Female territorial vigilance (arc-sine)^1^ | Male territorial vigilance (arc-sine) | Duet | 31.89 | < 0.0001 | 0.95 ± 0.09 | 0.93 (14) | < 0.0001 | 16 pairs |
|  |  | Female solo | 14.57 | 0.0001 | 0.73 ± 0.16 | 0.77 (14) | 0.0004 | 16 pairs |
|  |  | Male solo | 18.38 | 0.13 | 0.24 ± 0.17 | 0.49 (12) | 0.07 | 14 pairs |
|  |  | Control | 8.67 | 0.003 | 0.77 ± 0.20 | 0.76 (11) | 0.002 | 13 pairs |
| Female song rate^2^ | Male song rate | Duet | 4.74 | 0.03 | 0.39 ± 0.18 | 0.91 (14) | < 0.0001 | 16 pairs |
|  |  | Female solo | 9.10 | 0.003 | 0.37 ± 0.12 | 0.85 (14) | < 0.0001 | 16 pairs |
|  |  | Male solo | 1.27 | 0.26 | 0.16 ± 0.14 | 0.37 (14) | 0.15 | 16 pairs |
|  |  | Control | 2.89 | 0.09 | 0.38 ± 0.20 | 0.78 (14) | 0.0004 | 16 pairs |
| Female song duration^1^ | Male song duration | Duet | 15.44 | < 0.0001 | 0.71 ± 0.16 | 0.63 (30) | 0.0001 | 32 trials, 16 pairs |
|  |  | Female solo | 35.04 | < 0.0001 | 0.85 ± 0.10 | 0.81 (27) | < 0.0001 | 29 trials, 16 pairs |
|  |  | Male solo | 1.13 | 0.29 | 0.23 ± 0.22 | 0.30 (23) | 0.15 | 25 trials, 16 pairs |
|  |  | Control | 8.04 | 0.005 | 0.40 ± 0.13 | 0.70 (12) | 0.005 | 14 trials, 12 pairs |

^1^ Family: Gaussian; Random effects: stimulus identity. ^2^ Family: Poisson; Random effects: Stimulus identity. ^3^ Family: Gaussian; Random effects: stimulus identity, playback trial identity and group identity.
